# A Stability-Indicating Assay for Tetrahydrocurcumin-Diglutaric Acid and Its Applications to Evaluate Bioaccessibility in an In Vitro Digestive Model

**DOI:** 10.3390/molecules28041678

**Published:** 2023-02-09

**Authors:** Nattapong Jongjitphisut, Worathat Thitikornpong, Wisut Wichitnithad, Thanundorn Thanusuwannasak, Opa Vajragupta, Pornchai Rojsitthisak

**Affiliations:** 1 Pharmaceutical Sciences and Technology Program, Faculty of Pharmaceutical Sciences, Chulalongkorn University, Bangkok 10330, Thailand; 2 Government Pharmaceutical Organization, Bangkok 10400, Thailand; 3 Center of Excellence in Natural Products for Ageing and Chronic Diseases, Chulalongkorn University, Bangkok 10330, Thailand; 4 Department of Food and Pharmaceutical Chemistry, Faculty of Pharmaceutical Sciences, Chulalongkorn University, Bangkok 10330, Thailand; 5 Department of Analytical and Clinical Development, Pharma Nueva Co., Ltd., Bangkok 10900, Thailand; 6 CU Drug and Health Products Innovation Promotion Center, Faculty of Pharmaceutical Sciences, Chulalongkorn University, Bangkok 10330, Thailand; 7 Molecular Probes for Imaging Research Network, Faculty of Pharmaceutical Sciences, Chulalongkorn University, Bangkok 10330, Thailand

**Keywords:** tetrahydrocurcumin diglutaric acid (TDG), tetrahydrocurcumin (THC), UHPLC, validation, stability indicating assay, bioaccessibility

## Abstract

A simple and reliable ultra-high-performance liquid chromatographic (UHPLC) method was developed and validated for determination of tetrahydrocurcumin diglutaric acid (TDG) and applied for evaluation of its bioaccessibility. The analytical method was validated to demonstrate as a stability-indicating assay (SIA) according to the ICH Q2(R1) guidelines under various force degradation conditions including thermal degradation, moisture, acid and base hydrolysis, oxidation, and photolysis. The developed chromatographic condition could completely separate all degradants from the analyte of interest. The method linearity was verified in the range of 0.4–12 μg/mL with the coefficient of determination (r^2^) > 0.995. The accuracy and precision of the method provided %recovery in the range of 98.9–104.2% and %RSD lower than 4.97%, respectively. The limit of detection and quantitation were found to be 0.25 μg/mL and 0.40 μg/mL, respectively. This method has been successfully applied for the bioaccessibility assessment of TDG with the bioaccessibility of TDG approximately four fold greater than THC in simulated gastrointestinal fluid. The validated SIA method can also benefit the quality control of TDG raw materials in pharmaceutical and nutraceutical development.

## 1. Introduction

**Figure 1:**
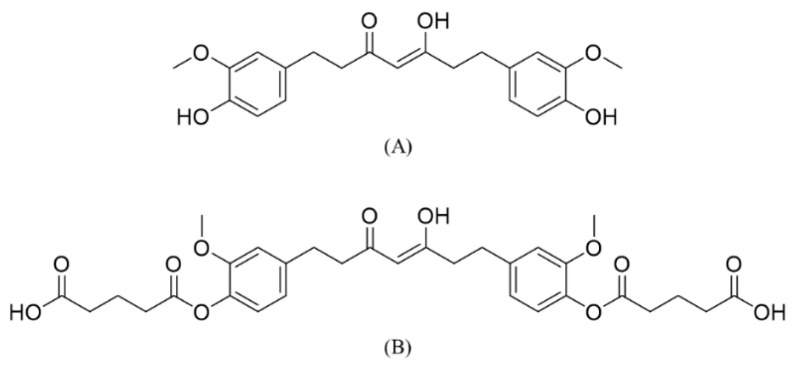
Chemical structures of (**A**) tetrahydrocurcumin (THC) and (**B**) tetrahydrocurcumin-diglutaric acid (TDG).

Curcumin (CUR) is one of the active substances derived from the turmeric plant (*Curcuma longa*) and has a wide range of biological activity. However, its pharmaceutical and nutraceutical applications have disadvantages due to chemical instability, poor absorption, rapid metabolism and rapid systemic elimination [1]. Several strategies have been used to overcome the limitations of CUR including chemical modification and prodrug and nanoformulation approaches [2,3,4,5,6,7,8]. In addition, tetrahydrocurcumin (THC, Figure 1A), an active metabolite of CUR, has been used as an alternative to CUR due to its better stability and some biological activities [9,10,11]. THC has been shown to exhibit antioxidant [12], anti-inflammatory [12,13,14], anticancer [12,15] and hepatoprotective activities [16,17,18]. THC possesses a liver protective function in in vitro and in vivo hepatotoxicity models induced by various drugs including acetaminophen [16], chloroquine [17] and erythromycin estolate [18]. THC is more effective than CUR in selectively inhibiting the expression of cyclooxygenase-2 (COX-2) and suppressing nuclear factor-κB (NF-κB) pathways [15]. Despite the superior antioxidant effects of THC [9,10] and better chemical stability [11] compared to CUR, therapeutic applications of THC were limited by its low systemic bioavailability due to poor bioaccessibility.

Bioaccessibility is defined as the amount of a target substance ready for absorption through a biological membrane and subsequently presented in systemic circulation. Therefore, the solubility in biological media and absorption through a biological membrane after oral administration are essential for assessing bioaccessibility [19]. Tang et al. (2021) synthesized and evaluated the bioaccessibility of tetrahydrocurcumin-hyaluronic acid conjugate (THC-HA) using in vitro and ex vivo models [20]. They found that mucoadhesive properties, in combination with the aqueous solubility enhancement, contributed to the increased bioaccessibility [20]. We recently synthesized tetrahydrocurcumin-diglutaric acid (TDG, Figure 1B) with enhanced hepatoprotective effects against alcohol-induced liver toxicity compared to THC [21], suggesting its potential use for the treatment of alcoholic liver disease (ALD) [21]. Structurally, as a diglutaric ester prodrug of THC, TDG was converted to its parent THC and subsequently exerted hepatoprotective effects.

The analytical methods for quantification of THC have been developed to determine the amount of THC in various matrices [22,23,24,25]. In 2005, high-performance liquid chromatography (HPLC) coupled with ultraviolet spectrophotometry (UV) was developed using a gradient elution mode with a total run time of 28 min [23]. Later, HPLC coupled with mass spectrometry (MS) [22,24,25] was developed. During the quantification method development of TDG for solubility, partition coefficient and kinetic studies [21], a simple analytical method for the simultaneous determination of TDG and THC in aqueous solutions and plasma was achieved. Nevertheless, this method has not been validated and proven to be a stability-indicating assay (SIA) for future implementation as a routine quantification of TDG in raw materials and other metrics.

TDG is a biodegradable prodrug that can release THC via tetrahydrocurcumin monoglutaric acid (TMG) intermediate. Therefore, a forced degradation approach has been used to generate all possible potential degradants of bioactive compounds to determine the separation capability of the developed analytical method. One of the specific aims of this study is to prove the suitability and validity of the developed method for routine quantitative analysis. Furthermore, forced degradation studies of TDG under various stress conditions, including acid/base hydrolysis, moisture hydrolysis, heat, light and oxidation, were performed to ensure the specificity and selectivity of the method, which can establish an SIA of the validated method for assay and drug stability monitoring. The validated SIA was also applied to the bioaccessibility evaluation of THC and TDG to demonstrate the method’s applicability in pharmaceutical development.

## 2. Results and Discussion

### 2.1. Method Development

The UHPLC analytical method was developed to quantify TDG in the samples for the evaluation of bioaccessibility, which is essential for future preclinical pharmacokinetic studies. Two potential impurities that can be found in TDG are THC and TMG. THC is a starting material for TDG synthesis, while TMG is considered as a potential by-product that occurred during esterification. In addition, TDG can be degraded to TMG and THC via ester hydrolysis. Therefore, both THC and TMG can be classified as known impurities. These unknown impurities can be obtained from the forced degradation experiments, in which the six stress conditions are used in this study. The objective of the chromatographic condition developed in this study is to demonstrate the separation capability of known and unknown degradation products from TDG. The chromatographic separation of TDG from its degradation products was modified from the method previously reported by Trivedi et al. (2020) [24]. The chromatographic parameters including mobile phase compositions and column types were optimized to achieve sufficient separation of TDG from other peaks. The best chromatographic result was achieved from a reversed-phase C18 (4.6 × 50 mm, 2.7 μm i.d.) column (Wilmington, DE, USA) and a mobile phase composed of 1% formic acid in water and 1% formic acid in acetonitrile (60:40) using an isocratic elution at a flow rate of 1.0 mL/min. The diluent was used as a mobile phase to obtain the best peak sharp without baseline drift. The maximum absorption of TDG at 280 nm was used as a detection wavelength. A short chromatographic run time of 9 min was achieved. The photodiode array was employed to ensure peak purity under forced degradation studies.

**Figure 2:**
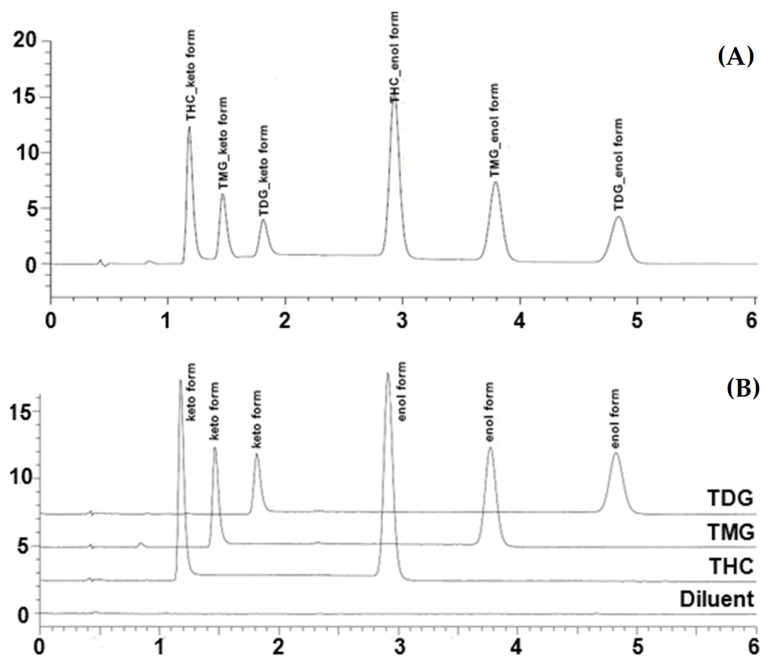
(**A**) The chromatogram of system suitability containing 10 μg/mL of THC, TMG, and TDG and (**B**) overlaid chromatograms of diluent, THC, and TDG standard solutions.

Structurally, curcumin can exhibit two tautomers as keto and enol forms. Several factors such as pH, solvent polarity, and temperature, have been reported to affect the equilibrium of keto-enol tautomerism [26]. In the NMR study, non-polar solvents such as chloroform-*d*_6_ (CDCl_3_) can promote the predominated enol form [27]. Similarly, TDG and THC can exhibit keto-enol tautomerism as curcumin. However, the keto tautomer is usually slightly presented in the ^1^H and ^13^C NMR studies as previously reported [21]. Under the chromatographic condition used in this study, the keto-enol tautomerization of analytes is noticeable even though the acidic and polar mobile phase was used (Figure 2A). The two tautomers of TDG are presented at 1.80 and 4.80 min for keto and enol forms, respectively. The THC tautomers are shown at 1.15 and 2.90 min for the keto and enol forms, respectively. Two tautomers are retained at 1.50 and 3.75 min for keto and enol forms of TMG, respectively. The ratio of enol and keto forms of TDG calculated from the calibration samples at the various concentrations is remarkably constant with the enol/keto peak ratio between 1.7 and 1.9, demonstrating the predominant enol form. In this study, the calibration curve and quantitative analysis of TDG, THC and TMG were calculated under the combination of peak areas of the two tautomers.

The keto-enol tautomerism of THC was not previously reported in the HPLC-UV method for quantification of THC in plasma [23]. In 2013, the HPLC-UV method was established for quantifying THC in biological metrics. The mobile phase composed of acetonitrile, methanol, and water (40:23:37) at pH 3.0 was used, and THC was eluted at 6.72 min without reporting the keto and enol forms of THC [28]. Raut and Shaji (2021) developed and validated the HPLC method for quantification of THC in bulk drugs and formulation [29]. However, this developed method has not been proven in terms of specificity via the force degradation experiments.

The keto-enol tautomerism of THC and its derivatives has been an issue in developing the analytical method for separating the tautomers. Our reported method demonstrated good separation of the four tautomers from TDG and THC with a short run time using the mobile phase compatible with most reversed-phase columns. The specificity and selectivity of the methodology have been proven and demonstrated in terms of specified and unspecified degradation products of TDG. In this regard, our developed UHPLC-UV method also provides the benefit of separating and providing a deep insight into the tautomer characteristics of THC and its derivatives. More importantly, the developed method is used not only for qualifying TDG in bulk drugs and its formulation but has also been applied in verifying in vitro kinetic [21] and bioaccessibility studies of TDG compared to THC.

### 2.2. Method Validation

#### 2.2.1. System Suitability

The tautomerization of TDG, THC and TMG individually shared keto and enol peaks at different retention times, presenting six separate peaks in the representative chromatogram as shown in Figure 2A. Overlaid separate injections of three compounds are demonstrated in Figure 2B. Regarding the system performance, the resolutions of two adjacent peaks were greater than 2.0, and the tailing factors of all representative peaks were less than 1.5. In addition, the average theoretical plate counts of TDG keto and enol forms were 2189 and 5608, respectively. Regarding the system reproducibility, %CVs of retention times and peak areas were in the range of 0.23–0.28 and 0.45, respectively. The system reproducibility parameters of TDG are summarized in Table 1.

#### 2.2.2. Specificity and Forced Degradation Studies

**Figure 3:**
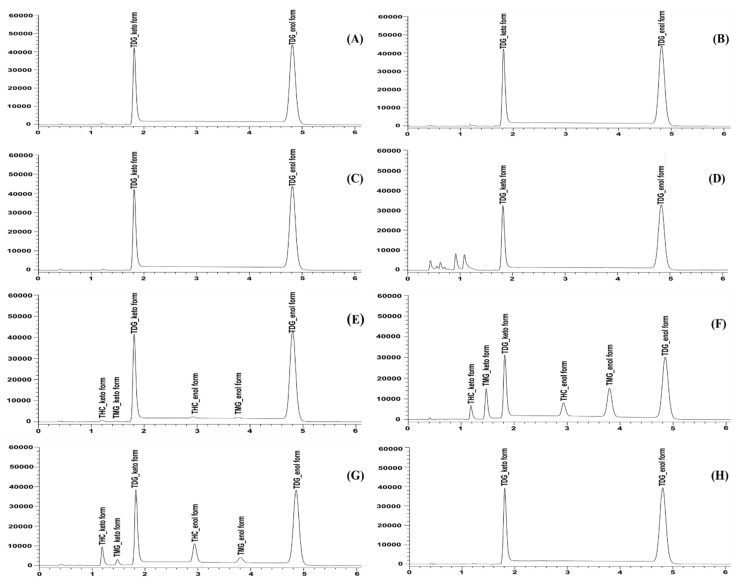
The chromatograms of forced degradation samples of tetrahydrocurcumin di-glutaric acid (TDG) under (**A**) photolysis (liquid), (**B**) photolysis (solid), (**C**) thermal stress, (**D**) oxidative stress, (**E**) moisture hydrolysis, (**F**) basic hydrolysis, (**G**) acid hydrolysis, and (**H**) control conditions.

Specificity is the ability of the method to discriminate an analyte from matrix components, in-process impurities, and degradation products. As shown in Figure 2, no interference was observed at the retention times of the TDG keto and enol forms. The chromatograms showed no coelution peaks of the known degradation products (THC and TMG) at the retention times of the TDG ketone and enol forms. According to Figure 3, the forced degradation results showed that TDG degraded under stress conditions to different extents. TDG was unstable, being hydrolyzed under both acid and basic conditions. The presented TMG and THC peaks were separated from the TDG peaks. THC was a major degradation product under acidic conditions, while TMG was predominant under basic conditions. Under oxidative stress, degradant peaks (R_t_ = 0.43–1.08 min) were found before the THC keto peak (Figure 3). Liquid chromatography coupled with triple quadrupole mass spectrometry (LC-MS/MS) was employed to characterize known and unknown degradants (See Supplementary Materials Section 4). All impurity peaks separated from the keto and enol peaks of TDG with a resolution greater than 2, indicating sufficient separation. The purity indices using the LabSolution client software for two tautomer forms of TDG were greater than their single-point thresholds for all stress conditions, as summarized in Table 2. The peak purity analysis indicated that the TDG peak had no coelution effect. The forced degradation results indicated that the developed method had sufficient specificity with good stability, indicating characteristics for the quantitative determination of TDG.

#### 2.2.3. Linearity and Range

Linearity was evaluated by analyzing the standard solution at various concentrations ranging from 0.4–12.0 μg/mL. Calibration curves were plotted from the concentrations of the analyte against the total peak response of the keto and enol forms. A plot between the concentrations versus the total peak responses of keto and enol TDGs is linear with a coefficient of determination (r^2^) of 0.9999. A linear regression analysis showed that the method was linear over the proposed concentration range.

The three-replicate calibration curve was verified using the unweighted linear model. The linearity equation was tested using back-calculated concentrations demonstrated in Table 3. The percentage of the relative error of mean back-calculated and actual concentrations of TDG was between −5.00 and 1.28. The %CV (*n* = 3) of the back-calculated concentration was less than 2.63. The residual plots and regression analysis generated using one-way analysis of variance (ANOVA) demonstrated that the *F* value (*F_table_*) of the regression line was significantly less than the calculated *F* value (*F_cal_*). The results indicate a good linear relationship between the peak response from the instrument (*y*) and the concentration of the analyte (*x*). Moreover, the *p*-value of the slope and *y*-intercept were calculated and the results were summarized in Table 3. The *p*-value of the slope was less than 0.05, indicating a significant difference versus zero. The *p*-value of the *y*-intercept exceeded 0.05, indicating that the intercepts of the regression line were insignificantly different from zero. Consequently, either a calibration curve or single-point calibration standard can be routinely applied in the TDG analysis.

#### 2.2.4. Limit of Detection (LOD) and Limit of Quantification (LOQ)

The LOD and LOQ for TDG analysis were experimentally determined, and the results showed that the concentration for LOD was 0.25 μg/mL (%CV = 4.1) with the S/N of 12, while the concentration for LOQ was 0.40 μg/mL with the S/N of 16. The % recovery was within 94.7–99.6% with the %CV of 1.8.

#### 2.2.5. Accuracy and Precision

Spiked quality control (QC) samples were prepared at LOQ, low, medium, and high concentration levels. The concentration levels of QC samples were distributed throughout the analysis range and used for accuracy and precision evaluation. The intra- and inter-day accuracy and precision were evaluated in triplicate at four levels of spiked QC samples, including 0.4 (LOQ), 8.0, 10.0, and 12.0 μg/mL (*n* = 3). The spiked LOQ QC samples for intra-day accuracy exhibited a %recovery from 99.3 to 104.6 with a %CV < 2.62. Regarding the inter-day evaluation, two-day accuracy demonstrated %recovery from 96.5 to 104.6 with %CV < 3.32. The spiked QC samples (80–120%) for intra-day and inter-day accuracy with precision exhibited %recovery ranging from 100.2 to 101.8 with %CV < 0.52 and 98.3 to 101.8 with %CV < 1.14, respectively. The results summarized in Table 4 demonstrated that the reliability of the proposed method was achieved.

#### 2.2.6. Robustness

To assess the robustness of the developed method, system performance was tested by injecting six replicates of the system suitability solution under the small variation in chromatographic conditions such as flow rate, column temperature and %formic acid in the mobile phase. The flow rate was varied in the range of ±0.1 mL/min from the proposed chromatographic condition, while the column temperature was varied by ± 2% from the proposed temperature. The concentration of the formic acid solution varied in the range of 0.1% from the proposed condition. The results of robustness are summarized in Table 5. In the case of flow rate variation, the retention times of keto and enol peaks ranged from 1.67 to 2.00 min with a %CV of < 0.30 and 4.44 to 5.33 min with a %CV of <0.17, respectively. The %CV of peak response was less than 0.48.

Regarding the effect of column temperature changes, the retention times of keto and enol peaks varied from 1.79 to 1.88 min with a %CV of < 0.30 and 4.72 to 5.03 min with a %CV of < 0.17, respectively. The %CV of peak response was less than 0.52. For the variation of %formic acid, the retention times of the keto and enol peaks ranged from 1.81 to 1.88 min with a %CV of < 0.30 and 4.78 to 5.05 min with a %CV of < 0.28, respectively. The %CV of peak response was less than 0.30. For all cases, the tailing factor was lower than 1.5, and the theoretical plates were greater than 2000. The results implied that the chromatographic condition provided sufficient robustness to the method.

### 2.3. In Vitro Digestion

In vitro digestion is a model for the assessment of bioaccessibility. The biological digestion processes mainly focus on three stages: the mouth, stomach, and small intestine. The biological fluids such as simulated saliva fluid (SSF), simulated gastric fluid (SGF) and simulated intestinal fluid (SIF) were therefore employed to evaluate the availability of the test substances after digestion. The bioaccessibility was calculated using the following equation:

(1) $$\%\mathrm{Bioaccessibility}=\frac{\mathrm{amount}\;\mathrm{of}\;\mathrm{sample}\;\mathrm{in}\;\mathrm{the}\;\mathrm{supernatant}}{\mathrm{amount}\;\mathrm{of}\;\mathrm{sample}\;\mathrm{before}\;\mathrm{digestion}}\;\times 100$$

**Figure 4:**
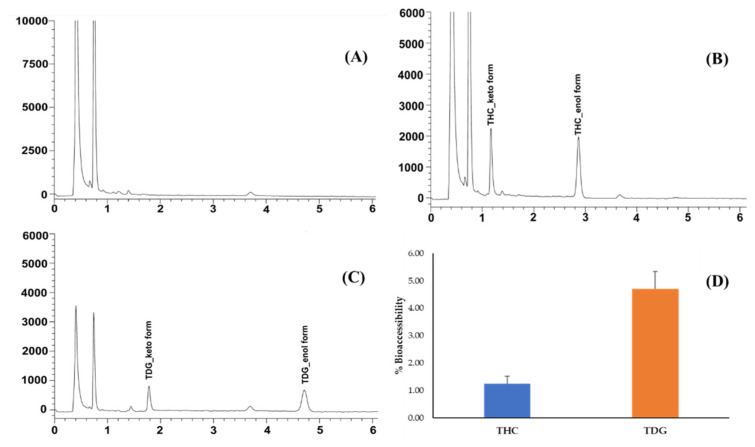
Representative chromatograms of (**A**) blank digestive enzyme, (**B**) THC and (**C**) TDG after digestion; (**D**) %Bioaccessibility of THC and TDG (mean ± SD, *n* = 3).

The results showed that the validated UHPLC could separate THC and TDG from simulated gastrointestinal matrices (Figure 4A–C). The peak purity indices for both peaks are greater than 0.99 (See Supplementary Materials Figure S2), indicating no coelution of matrices or unspecified degradation on the analyte peaks. The greater amount of TDG in the simulated gastrointestinal fluids indicated that TDG was more available than THC for absorption (Figure 4D). The solubility profile of TDG, especially in essential solutions, may contribute to an improved bioaccessibility of TDG [21]. Moreover, THC and TDG were stable after incubation in the SIF at 37 °C for 2 h. The development of drug candidates with better aqueous solubility is a dominant strategy to improve bioaccessibility, which may enhance drug bioavailability, resulting in greater pharmacological activities. Previously, the designed prodrug of curcumin conjugated with glutaric acid demonstrated greater solubility than curcumin [6]. Moreover, its anti-inflammatory effect was also improved in the preclinical study [30]. Further in vivo pharmacokinetic studies should be performed to confirm the potential of TDG with regard to oral bioavailability improvement.

## 3. Materials and Methods

### 3.1. Chemicals and Reagents

THC (lot no. ZL20180816, purity 99.08%) was obtained from the Zhonglan industry (Zhonglan Industry, Shandong, China). TDG (purity 99.71%) was synthesized and characterized as previously reported [21]. Tetrahydrocurcumin-monoglutaric acid (TMG) was synthesized and assayed for purity (see Supplementary Materials, Figure S1). All reagents were at least of analytical grade and were procured from commercial sources. Formic acid was obtained from Carlo Erba (Val de Reuil, France). The HPLC grade of acetonitrile (ACN) was purchased from Fisher Scientific (Soul, South Korea). Hydrochloric acid and sodium hydroxide were obtained from QRëc (Auckland, New Zealand) and Carlo Erba (Val de Reuil, France), respectively. Bile, bovine serum albumin, lipase, mucin, pancreatin, α-amylase, and pepsin were obtained from Sigma-Aldrich Co., Ltd. (St. Louis, MO, USA). Calcium chloride anhydrous powder was purchased from Carlo-Erba (Val de Reuil, France). The ultrapure water (18.2 MΩ-cm) used throughout the study was produced in-house using a Milli-Q^®^ integral water purification system (Millipore, S.A.S, France).

### 3.2. Instrumentations and Conditions

Samples were analyzed using a Prominence UHPLC system (Shimadzu, Kyoto, Japan). Chromatographic separation was performed on a HALO C18 (4.6 × 50 mm, 2.7 mm i.d) column (Wilmington, DE, USA) under an isocratic elution at a flow rate of 1.0 mL/min. The mobile phase consists of 1% formic acid in water and 1% formic acid in ACN (60:40). The temperatures of the column and autosampler were maintained at 35 °C and 25 °C, respectively. The detection wavelength was set at 280 nm. The injection volume was 10 µL. The total analysis time was 9 min per injection. Acquisition and processing of data were operated using the LabSolutions CS^TM^ (version 6.86SP2, Shimadzu, Kyoto, Japan).

### 3.3. Preparation of Standard and System Suitability Solutions

Standard stock solutions of TDG, TMG and THC (100 μg/mL) were separately prepared by dissolving 5 mg of each compound with ACN in a 50-mL volumetric flask. A 5 mL of standard stock solution of TDG was transferred to another 50-mL volumetric flask and further diluted to volume with a mobile phase (60:40 of 1% formic acid in water: 1% formic acid in ACN) to obtain a working standard solution of TDG at the concentration of 10 μg/mL. Standard solutions of TMG and THC at the same concentration were prepared similarly to the working standard of THG. The standard solutions were then filtered through 0.45 μm nylon membrane filters before analysis.

A system suitability solution was prepared by diluting the standard stock solutions of THG, THC, and TMG (100 μg/mL) with the mobile phase to obtain a solution containing 10 μg/mL each. The suitability solution was filtered through a 0.45 μm nylon membrane filter before analysis.

### 3.4. Forced Degradation Studies

Forced degradation studies of TDG under six stress conditions were performed as per the ICHQ1A(R2) guideline [31]. The sample was prepared at the nominal concentration of 100 μg/mL and filtered through a 0.45 μm nylon membrane filter before analysis.

#### 3.4.1. Procedure for Acid Hydrolysis

An amount of 5 mg of TDG was added into a 15-mL glass-stopper test tube followed by the addition of 200 μL of 1 N HCl in a 15-mL glass-stopper test tube. The mixture was vigorously vortexed until homogeneously dispersed. The sample was incubated at 80 °C for 6 h in a heating block dry bath. Next, 200 μL of 1 N NaOH was added to neutralize the remaining acid residue. After that, the neutralized sample was further dissolved with 5 mL of ACN. The resulting solution was transferred to a 50-mL volumetric flask and adjusted to the volume with the mobile phase.

#### 3.4.2. Procedure for Basic Hydrolysis

An amount of 5 mg of TDG was dispersed in 200 µL of 0.1 NaOH in a 15-mL glass-stopper test tube. The mixture was vigorously vortexed until it was homogeneously dispersed. The sample was incubated at ambient temperature for 3 h. Next, 200 μL of 0.1 N HCl was added to neutralize the remaining hydroxide residue. After that, the sample was further dissolved with 5 mL of ACN. The resulting solution was transferred to a 50-mL volumetric flask and made up the volume with the mobile phase.

#### 3.4.3. Procedure for Moisture Hydrolysis

A total of 5 mg of TDG was dispersed in 200 μL of purified water (Milli-Q) in a 15-mL glass-stopper test tube. The mixture was vigorously vortexed until a homogeneous mixture was achieved. The sample was incubated at 80 °C for 6 h. After that, the remaining sample was dissolved with 5 mL ACN. The resulting sample was transferred to a 50-mL volumetric flask and diluted to volume with the mobile phase.

#### 3.4.4. Procedure for Oxidative Degradation

An amount of 5 mg of TDG was mixed with 200 μL of 3% freshly prepared H_2_O_2_ in a 15-mL glass-stopper test tube and subsequently incubated at 80 °C. After 3 h, the sample residue was dissolved with 5 mL ACN and transferred to a 50-mL volumetric flask. The transferred solution was diluted to volume with the mobile phase.

#### 3.4.5. Procedure for Thermal Degradation

A total of 5 mg TDG was directly heated at 80 °C for 8 h in a 15-mL glass-stopper test tube. The sample was then dissolved with 5 mL of ACN, transferred to a 50-mL volumetric flask, and adjusted to volume with the mobile phase.

#### 3.4.6. Procedure for Photolysis

The photostability of TDG was investigated in both solid and solution states according to the ICH Q1B guideline [32]. A 2%*w/v* aqueous solution of quinine monohydrochloride dihydrate in a 1-cm quartz cell was used as a light intensity indicator. The quinine solution stood beside the test sample during the experiment. The study is appropriately valid when a change in UV absorbance of at least 0.5 at 400 nm is obtained. Based on the ICH Q1B, the overall illumination on the sample is greater than the 200-watt h/m^2^. For the photostability of TDG as the solid substance, 5 mg of TDG was placed in a photostability chamber with direct exposure to the fluorescent and UV light at room temperature until the UV absorbance deviation of the quinine solution at 400 nm was at least 0.5 of the absorbance unit. The test sample was then dissolved in 5 mL ACN and diluted with the mobile phase to obtain the final concentration of 100 μg/mL. For the photostability of TDG in solution, the standard stock solution of TDG in a 1-cm quartz cell was placed in a photostability chamber with direct exposure to the fluorescent and UV light at room temperature. The experiment was terminated under the same criteria as the solid sample. Subsequently, the irradiated solution was diluted with the mobile phase to obtain a sample solution at the concentration of 100 μg/mL.

### 3.5. Method Validation

The method was validated according to the ICH Q2(R1) guideline for the validation of analytical procedures in the aspect of assay procedures [33].

#### 3.5.1. System Suitability

The system suitability test was performed to verify the reproducibility and performance of the system. For system reproducibility, the working standard solution of TDG at concentrations of 10 μg/mL was injected in five replicates. The variation of system reproducibility was evaluated in terms of %CV of peak area and retention time.

The mixture solution of TDG, THC and TMG at the concentration of 10 μg/mL for each component was prepared and immediately used as a resolution solution to verify the system performance. The resolutions between two adjacent peaks were determined. Additionally, the tailing factor and the theoretical plate count of TDG were calculated to assess the performance of the chromatographic system.

#### 3.5.2. Specificity

Based on the synthetic route of TDG, TMG and THC are identified as potential in-process impurities in TDG raw materials. More importantly, TDG can degrade to TMG and even THC under inappropriate storage conditions. Consequently, those were included as potential known impurities for evaluating the specificity and selectivity of the optimized chromatographic system. The specificity of the developed method was assessed by separately injecting the mobile phase (diluent), TDG standard solution (10 μg/mL), THC standard solution (μg/mL), and TMG standard solution (10 μg/mL), resolution solution, and the forced degradation samples.

#### 3.5.3. Linearity and Range

Linearity describes the ability of the analytical method to reveal the proportional expression between the response signals from the instrument and the concentration of analyte in the sample within a specified dynamic range. The range of the method is defined as the concentration interval between the upper and lower quantitative levels. A serial dilution of TDG standard solutions at the concentrations between 0.4 and 12 μg/mL was prepared by diluting appropriate volumes of the standard stock solution of TDG (100 μg/mL) with the mobile phase. Linearity and range were established by plotting the total peak response of keto and enol forms against their concentration. The slope, intercept, and coefficient of determination (r^2^) of the calibration curve were calculated.

#### 3.5.4. Limit of Detection (LOD) and Limit of Quantitation (LOQ)

The LOD is defined as the lowest amount of an analyte in a sample that is detectable but not necessarily quantifiable as an exact value. The LOD can be derived from the signal-to-noise (S/N) ratio, which is typically expressed as the analyte concentration. The standard stock solution of TDG was serially diluted with the mobile phase to investigate a LOD concentration. The LOD was theoretically verified from the S/N ratio of ≥3 and %CV of five replicate injections of ≤15.

The LOQ is expressed as the lowest amount of analyte in a sample with an S/N ratio of ≥10 that can be quantitatively analyzed with appropriate accuracy and precision. The standard stock solution of TDG was serially diluted with the mobile phase to investigate the LOQ concentration. The accuracy and precision were accepted at a % recovery of 80–110% and %CV of <11%, respectively.

#### 3.5.5. Accuracy

The accuracy of the analytical procedure expresses the closeness of added and found amounts. The accuracy of TDG analysis was determined using the TDG standard solution prepared by diluting appropriate volumes of the standard stock solution of TDG with the mobile phase to obtain the final concentration of 0.4, 8, 10, and 12 μg/mL, which are represented to LOQ, 80, 100, and 120% nominal concentration, respectively. Each concentration was prepared for three replicates, and each replicate was injected in triplicate. Accuracy was performed by the spiked standard method, and the evaluation was expressed as %recovery.

#### 3.5.6. Precision

The precision of the analytical method is usually evaluated in aspects of repeatability and intermediate precision which are typically expressed as the coefficient of variation of the multiple measurements at the same concentration of the homogeneous sample. For repeatability, it is the with-run precision determined using triplicate samples of TDG at the concentrations of 0.4, 8, 10, and 12 μg/mL, representing LOQ, 80, 100, and 120% nominal concentration, respectively, as described in accuracy. For intermediate precision, the samples of TDG at the concentrations of 0.4, 9, 10 and 12 μg/mL were prepared in the same manner as repeatability but assayed on two different days and by two different analysts. The %CVs of each concentration for two different precision experiments were evaluated.

#### 3.5.7. Robustness

Method robustness was verified under small variations in method parameters, including the changes in mobile phase ratio, flow rate and column temperature. Suitability parameters including retention time, peak response, tailing factor, and a theoretical plate of TDG were observed when method parameters were varied from regular method conditions.

### 3.6. In Vitro Digestion

The in vitro digestion model was slightly modified from the previous report by Zhou et al. (2018) [34]. Various synthetic fluids used in the in vitro human digestive model were prepared as described by Wang et al. (2019) [35]. The composition of digestive enzymes including saliva, gastric and duodenal juices and bile acid is summarized in the Supplementary Materials Section 2.

Fifty milligrams of THC and TDG were added in 3 mL simulated salivary fluid (SSF) and then adjusted to pH 6.8. The solution was incubated at 37 °C in a shaking bath at 100 rpm for 5 min. Next, 6 mL of preheated simulated gastric fluid (SGF) was added to the solution mixture, adjusted to pH 2.0 and incubated at 37 °C for 2 h. After that, 6 mL of preheated simulated intestinal fluid (SIF) and 3 mL of bile juice were added to the solution mixture, adjusted to pH 6.8, and incubated at 37 °C for 2 h. After incubation, the digestion mixture was collected and centrifuged at 5500 rpm, 25 °C for 10 min. Two hundred microliters of supernatant were added to 200 μL of 1% formic acid to stop enzyme activity. Subsequently, 400 μL of ACN was added to the mixture to precipitate the protein and extract target analytes. The solution mixture was then centrifuged at 14,000 rpm at 25 °C for 10 min. THC and TDG samples were diluted with the mobile phase at dilution factors of 5 and 25, respectively. The sample was analyzed using the validated UHPLC method. To ensure no residue protein matrix coelution with TDG and THC, the PDA detector was employed to evaluate the peak purity of TDG and THC peaks.

## 4. Conclusions

A reliable and reproducible stability-indicating UHPLC method was developed for the quantitative analysis of TDG. Forced degradation studies demonstrated that TDG was degraded under acid and basic conditions to different extents. The chromatographic condition can sufficiently provide the specificity of the method with a great peak purity index. The method was also fully validated according to the ICH guideline Q2 (R1). The validated method was successfully applied to evaluate the bioaccessibility of TDG and THC in simulated gastrointestinal fluids.

## Figures and Tables

**Table 1:** System suitability parameters of TDG (*n* = 5).

Injection No.	Retention Time (min)		Peak Response (sum)	Tailing Factor		Theoretical Plates		Resolution	
	Keto Form	Enol Form		Keto Form	Enol Form	Keto Form	Enol Form	Keto Form	Enol Form
1	1.812	4.839	55,937	1.271	1.012	2192	5462	2.41	4.41
2	1.803	4.816	56,079	1.304	1.027	2156	5576	2.42	4.45
3	1.798	4.809	55,885	1.319	1.028	2234	5711	2.42	4.42
4	1.806	4.824	55,945	1.329	1.037	2218	5679	2.44	4.48
5	1.803	4.822	55,427	1.294	1.043	2146	5611	2.40	4.46
Mean	1.804	4.822	55,855	1.303	1.029	2189	5608	2.42	4.44
%CV	0.28	0.23	0.45	-	-	-	-	-	-

**Table 2:** Peak purity values of tetrahydrocurcumin di-glutaric acid (TDG) under stress conditions.

Treatment	Peak Purity Index (Keto)	Peak Purity Index (Enol)	Single Point Threshold
Control (untreated)	0.999999	0.999999	0.999787
Acid hydrolysis (1.0 N HCl) at 80 °C for 6 h	0.999999	0.999999	0.999703
Basic hydrolysis (0.1 N NaOH) at room temperature for 3 h	1.000000	1.000000	0.998026
Moisture hydrolysis at 80 °C for 6 h	1.000000	0.999999	0.998911
Oxidative stress (3% H_2_O_2_) at 80 °C 3 h	0.999999	0.999999	0.998222
Thermal degradation at 80 °C for 8 h	0.999999	0.999998	0.998944
Photolysis in a solid state, 1.2 million lux h for 5 d	1.000000	1.000000	0.999067
Photolysis in solution, 1.2 million lux h for 5 d	1.000000	1.000000	0.999407

**Table 3:** Mean inter-day back-calculated standard and calibration curve results (*n* = 3).

Compound	Nominal Conc (μg/mL)	Back-Calculated Concentration (μg/mL)			Mean Back-Calculated Concentration (μg/mL) ± SD	%RE	%CV
		Day 1	Day 2	Day 3			
TDG	0.40	0.39	0.38	0.37	0.38 + 0.01	−5.00	2.63
	1.00	1.00	1.01	0.99	1.00 + 0.01	0.00	1.00
	2.00	1.94	2.00	1.99	1.98 + 0.03	−1.17	1.63
	4.00	3.91	3.97	4.05	3.98 + 0.07	−0.58	1.77
	6.00	6.02	6.01	6.20	6.08 + 0.11	1.28	1.76
	8.00	8.00	7.83	8.01	7.95 + 0.10	−0.67	1.27
	10.00	9.82	9.85	10.18	9.95 + 0.20	−0.50	2.01
	12.00	11.84	12.04	11.99	11.96 + 0.10	−0.36	0.87
	r^2^	0.9998	0.9997	0.9996			
	*F_cal_*	44,580.27635					
	*F_table_*	7.0732 × 10^−38^					
*p*-value	Slope	7.0732 × 10^−38^					
	*y*-intercept	0.9999					

**Table 4:** The accuracy and precision of the method.

Nominal Conc. (µg/mL)	Intra-Day (*n* = 3)				Inter-Day (*n* = 9)			
	Added Conc. (µg/mL)	Found Conc. (µg/mL)	%Recovery	%CV	Added Conc. (µg/mL)	Found Conc. (µg/mL)	%Recovery	%CV
0.4	0.40	0.41 ± 0.01	101.8	2.62	0.40	0.39 ± 0.01	98.6	3.32
8.0	7.99	8.07 ± 0.01	101.0	0.06	7.99	8.02 ± 0.02	100.4	0.30
10.0	9.99	10.07 ± 0.05	100.8	0.52	9.99	10.08 ± 0.02	100.9	0.15
12.0	11.99	12.16 ± 0.05	101.5	0.35	11.99	11.94 ± 0.14	99.6	1.14

**Table 5:** The robustness of the method (*n* = 5).

Chromatographic Parameters	Data Type	TDG Standard Solution						
		Retention Time		Peak Response	Tailing		Theoretical Plate	
		Keto Form	Enol Form		Keto Form	Enol Form	Keto Form	Enol Form
Flow rate								
0.9 mL/min	AVG	2.008	5.33	53,192	1.34	1.04	4134	7666
	%CV	0.17	0.06	0.48	-	-	-	-
1.0 mL/min	AVG	1.821	4.83	50,355	1.34	1.05	3886	7422
	%CV	0.30	0.17	0.30	-	-	-	-
1.1 mL/min	AVG	1.667	4.44	47860	1.34	1.04	3717	7091
	%CV	0.24	0.13	0.42	-	-	-	-
Column temperature								
33 °C	AVG	1.879	5.03	53,148	1.32	1.05	3957	7499
	%CV	0.19	0.17	0.25	-	-	-	-
35 °C	AVG	1.821	4.83	50,355	1.34	1.05	3886	7422
	%CV	0.30	0.17	0.30	-	-	-	-
37 °C	AVG	1.786	4.72	47,082	1.36	1.04	3925	7433
	%CV	0.20	0.08	0.52	-	-	-	-
Formic acid concentration								
0.9%	AVG	1.811	4.78	50,695	1.31	1.03	4172	7923
	%CV	0.16	0.07	0.28	-	-	-	-
1.0%	AVG	1.821	4.83	50,355	1.34	1.05	3886	7422
	%CV	0.30	0.17	0.30	-	-	-	-
1.1%	AVG	1.875	5.05	49,481	1.34	1.03	4253	7960
	%CV	0.14	0.07	0.12	-	-	-	-

## Data Availability

Not available.

## Supplementary Materials

The following supporting information can be downloaded at: https://www.mdpi.com/article/10.3390/molecules28041678/s1, Figure S1: ^1^H-NMR spectrum of tetrahydrocurcumin-monoglutaric acid (TMG); Figure S2: Peak purity index values of (A) TDG (B) THC in digestive samples; Figure S3: Representative chromatograms obtained from (A) THC and (B) TDG reference standards; Figure S4: Representative chromatograms obtained from (A) basic hydrolysis and (B) oxidative stress of TDG reference standard; Figure S5: Representative chromatograms obtained from (A) blank digestive enzymes, (B) THC in digestive enzymes, and (C) TDG in digestive enzymes; Figure S6: Representative chromatograms demonstrating the extract ion (XIC) at m/z 100-400 of the oxidative stress sample; Table S1: The Composition of Digestive Enzymes.

## Sample Availability

Samples of the compounds are not available from the authors.
