# Supplementary material for: A Stability-Indicating Assay for Tetrahydrocurcumin-Diglutaric Acid and Its Applications to Evaluate Bioaccessibility in an In Vitro Digestive Model
Source: Molecules. 2023 Feb 9;28(4):1678. doi: 10.3390/molecules28041678 (PMC9966976; doi:10.3390/molecules28041678)
Supplement: Supplementary file 1 [file molecules-28-01678-s001.zip › molecules-2097211-supplementary.pdf]

molecules

MDPI

Supplementary Materials

A Stability-Indicating Assay for Tetrahydrocurcumin-Diglutaric Acid and Its Applications to Evaluate Bioaccessibility in an In Vitro Digestive Model

Nattapong Jongjitphisut [1,2], Worathat Thitikornpong [3,4,*], Wisut Wichitnithad [5], Thanundorn Thanusuwannasak [6], Opa Vajragupta [3,7] and Pornchai Rojsitthisak [3,4]

1 Pharmaceutical Sciences and Technology Program, Faculty of Pharmaceutical Sciences, Chulalongkorn University, Bangkok 10330, Thailand
2 Government Pharmaceutical Organization, Bangkok 10400, Thailand
3 Center of Excellence in Natural Products for Ageing and Chronic Diseases, Chulalongkorn University, Bangkok 10330, Thailand
4 Department of Food and Pharmaceutical Chemistry, Faculty of Pharmaceutical Sciences, Chulalongkorn University, Bangkok 10330, Thailand
5 Department of Analytical and Clinical Development, Pharma Nueva Co., Ltd., Bangkok 10900, Thailand
6 CU Drug and Health Products Innovation Promotion Center, Faculty of Pharmaceutical Sciences, Chulalongkorn University, Bangkok 10330, Thailand
7 Molecular Probes for Imaging Research Network, Faculty of Pharmaceutical Sciences, Chulalongkorn University, Bangkok 10330, Thailand
* Correspondence: worathat.t@pharm.chula.ac.th; Tel.: +66-2-218-8315; Fax: +66-2-254-5195

Citation: Jongjitphisut, N.; Thitikornpong, W.; Wichitnithad, W.; Thanusuwannasak, T.; Vajragupta, O.; Rojsitthisak, P. A Stability-Indicating Assay for Tetrahydrocurcumin-Diglutaric Acid and Its Applications to Evaluate Bioaccessibility in an In Vitro Digestive Model. *Molecules* **2023**, *28*, 1678. https://doi.org/10.3390/molecules28041678

Academic Editor: Maria Z. Tsimidou

Received: 29 November 2022
Revised:31 January 2023
Accepted: 7 February 2023
Published: 9 February 2023

Copyright: © 2023 by the authors. Licensee MDPI, Basel, Switzerland. This article is an open access article distributed under the terms and conditions of the Creative Commons Attribution (CC BY) license (https://creativecommons.org/licenses/by/4.0/).

1. Synthesis of Tetrahydrocurcumin-Monoglutaric Acid (TMG)

Tetrahydrocurcumin (THC) (1.00 g, 2.69 mmol) and *N*, *N*-Diisopropylethylamine (DIPEA) (0.18 mL, 1.03 mmol) were dissolved in anhydrous dichloromethane (5 mL). The mixture was stirred under a nitrogen atmosphere at room temperature. At the same time, the solution of glutaric anhydride (0.30 g, 2.63 mmol) was gradually added to the mixture of THC and DIPEA for 1 h. The reaction was monitored by thin-layer chromatography (TLC). The reaction mixture was applied on a TLC plate, and the developing solvent consisted of hexane, dichloromethane and acetone (4:4:2, respectively) and 1% formic acid. After appearing a spot between TDG and THC, which was assumed as tetrahydrocurcumin-monoglutaric acid (TMG), the reaction was terminated. The reaction mixture was then washed with 0.1 M HCl and water. The dichloromethane portion was dried over anhydrous sodium sulfate and evaporated using a rotary evaporator. The residue was purified using column chromatography using mobile phases composed of hexane, dichloromethane, and acetone at various ratios (1:0:0 to 3:1:1) in the presence of 1% formic acid to obtain yellow viscous residue. The residue was characterized by ^{1}H NMR spectroscopy and elucidated as TMG (Figure S1). The product was subsequently used for ultra-performance liquid chromatography (UPLC) identification.

^{1}H NMR (500 MHz, $CDCl_3$) δ (ppm): 2.03–2.12 (m, 2H), 2.52–2.59 (m, 5H), 2.08 (t, J = 7.2 Hz, 2H), 2.74–2.91 (m, 5H), 3.52 (s, 1H), 3.78 (s, 3H), 3.84 (s, 3H), 5.43 (s, 1H), 6.62–6.82 (m, 5H), 6.90-6.93 (m, 1H).

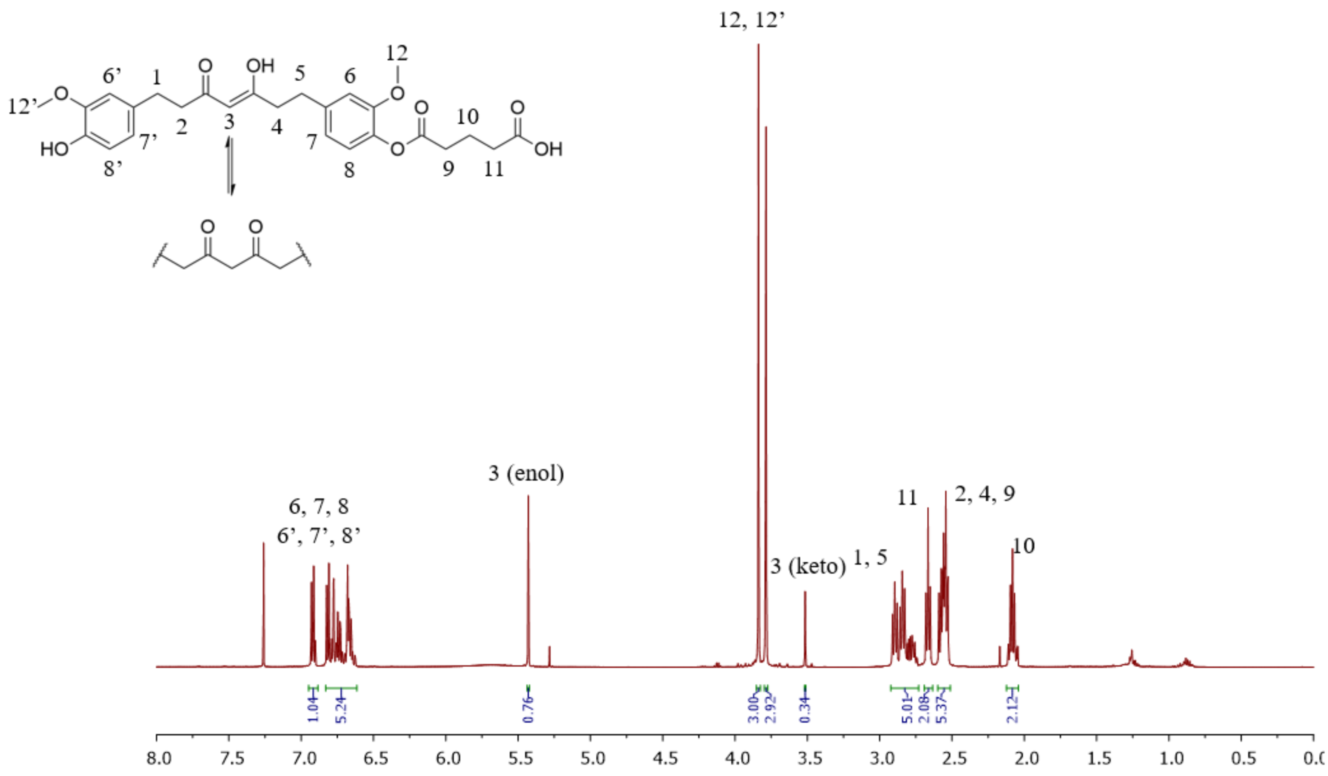

Figure S1. ^{1}H-NMR spectrum of tetrahydrocurcumin-monoglutaric acid (TMG).

2. The Composition of Digestive Enzymes

Table S1. The Composition of Digestive Enzymes.

**Simulated Saliva Fluid**	**Simulated Gastric Fluid**	**Simulated Intestinal Fluid**	**Bile Juice**
Sodium chloride 298 mg	Hydrochloric acid 24 mg	Potassium chloride 56.4 mg	Sodium bicarbonate 0.5785 g
Urea 20 mg	Calcium chloride dihydrate 40 mg	Calcium chloride dihydrate 20 mg	Calcium chloride dihydrate 22.2 mg
Uric acid 1.5 mg	BSA 0.1 g	BSA 0.1 g	BSA 0.1 g
α-amylase 29 mg	Pepsin 0.25 g	Pancreatin 0.9 g	Bile (Cholic acid) 3 g
Mucin 2.5 mg	Mucin 0.3 g	Lipase 0.15 g	-
Water 100 mL	Water 100 mL	Water 100 mL	Water 100 mL
Adjust pH to 6.8	Adjust pH to 2.0	Adjust pH to 8.0	Adjust pH to 7.0

Remark: preincubated enzyme at 37 °C prior to use.

3. Peak Purity indices of TDG and THC in Digestive Samples

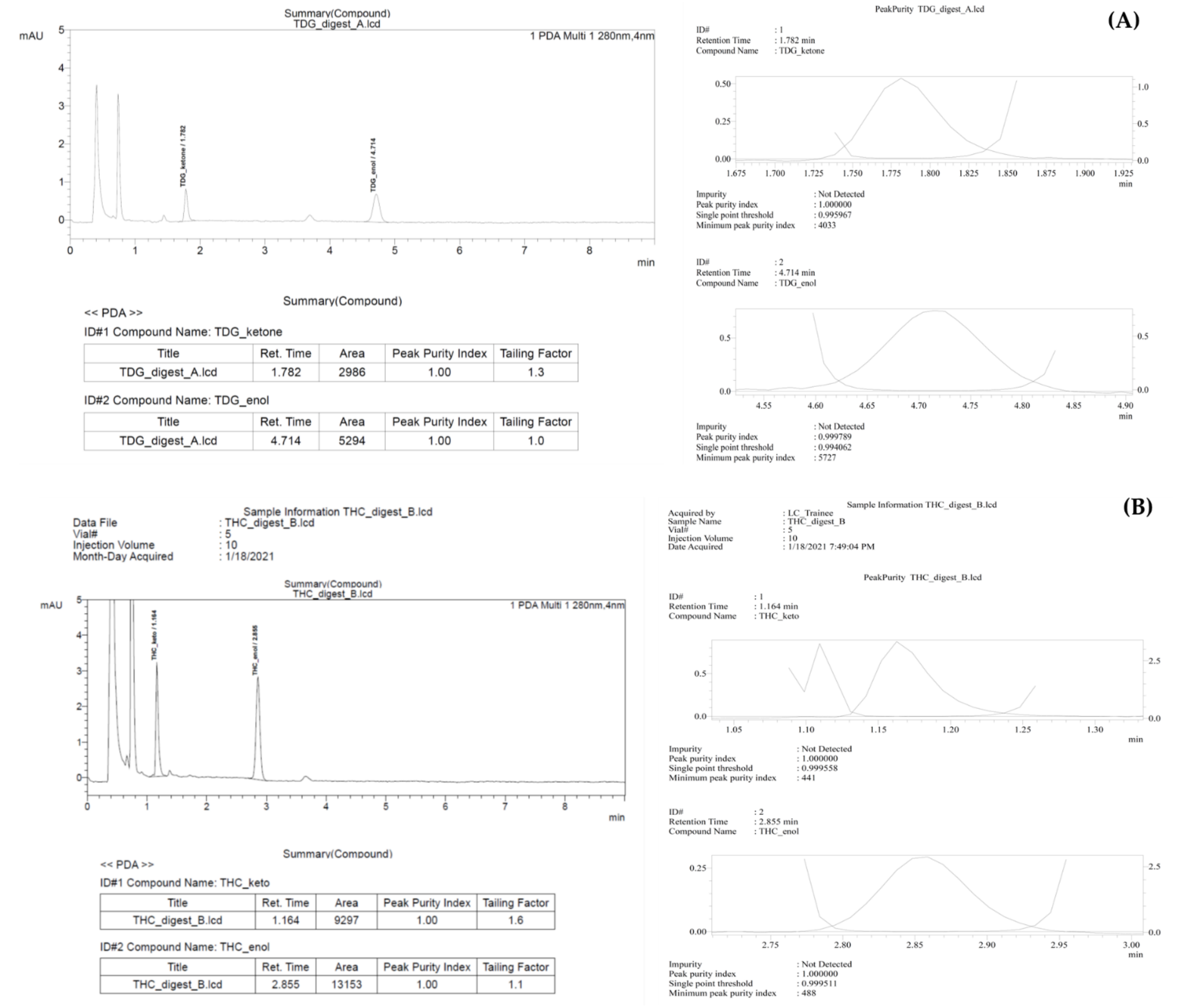

Figure S2. Peak purity index values of **(A)** TDG **(B)** THC in digestive samples.

4. Liquid Chromatography-Tandem Mass Spectrometry (LC-MS/MS) for Identification

4.1. Instrumentation and HPLC-MS/MS Conditions

The chromatographic separation of THC and TDG was carried out on a Prominence LC system (Shimadzu, Japan) equipped with a binary pump, an autosampler, and a column oven. Chromatographic separation was performed on a HALO C18 (4.6 × 50 mm, 2.7 mm i.d.) column (Wilmington, DE, USA) under an isocratic elution at a flow rate of 0.5 ml/min. The mobile phase consists of 1% formic acid in water and 1% formic acid in ACN (60:40). The temperatures of the column and autosampler were maintained at 35 °C and 25 °C, respectively. The injection volume was 10 µL. The total analysis time was 12 min per injection.

Mass spectrometric analysis was achieved with MS/MS detection in a positive ion mode using an AB Sciex API-4000 mass spectrometer (Foster City, CA, USA) equipped with a Turbo ionspray™ interface which was set at 450 °C. The ion spray voltage was set

at 1500 V. The source parameters including the collision gas, curtain gas, ion source gas 1, and ion source gas 2 were set at 5, 20, 60, and 50 psi, respectively. Nitrogen was designed as carrier and fragmentation gas. The MS parameters including the declustering potential (DP), entrance potential (EP), collision energy (CE), and collision cell exit potential (CXP) were set at 75, 10, 26, and 16 V, respectively, for THC, and 116, 10, 35, and 24 V, respectively, for TDG. Detection of the ions was carried out in the multiple reaction monitoring mode (MRM) by monitoring the mass transition at *m*/*z* [M + H^+] 373.20/137.10 for THC and *m*/*z* [M + Na^+] 623.30/509.30 for TDG with a 250 msec scan dwell time for all compounds. The data acquisition was processed using Analyst software™ (version 1.4.2).

4.2. Results and Discussion

In the force degradation and digestive studies, TMG and THC are represented as two identified degradation products of TDG. The Q1 MS showed predominated precursor ions at *m*/*z* 373.20 [M + H^+] and 623.30 [M + Na^+] for THC and TDG, respectively. After MS fragmentation, the most prominent and stable product ions for identification of THC and TDG were found at *m*/*z* 137.10 and 509.30, respectively. The chromatograms of THC and TDG reference standards were demonstrated in Figure S3A,B, respectively. Under the chromatographic condition, THC was eluted at retention times of 2.13 and 5.51 min for keto and enol forms, respectively. TDG was eluted at retention times of 3.50 and 9.79 min for keto and enol forms, respectively. The result presented that the TDG reference standard exhibited some in-source fragmentation, which showed up at mass transition of *m*/*z* 373.20/137.10 at the same retention times of TDG. In force degradation experiments, TDG is degraded to TMG and THC under basic hydrolysis (see Supplementary Information Figure S4A). THC mass transition (*m*/*z* 373.20/137.10) presented six peaks. THC keto and enol forms degraded from TDG were eluted at 2.13 and 5.51 min, respectively. THC keto and enol peaks were eluted at 3.50 and 9.79 min, respectively, derived from the in-source fragmentation of TDG. More interestingly, two additional THC peaks at the retentions of 2.74 and 7.37 min were found in the THC mass transition, which was derived from the in-source fragmentation of TMG. In contrast, the oxidative stress experiment found no THC peaks in the THC mass transition, except THC peaks at the retention times of 3.50 and 9.77 min derived from the in-source fragmentation of TDG (see Supplementary Information Figure S B). The result indicated that the TDG was not degraded to TMG and THC under oxidative stress.

For digestive samples, the representative chromatograms of blank, THC, and TDG in digestive enzymes are demonstrated in Figure S5A–C. Regarding TDG in the presence of digestive enzymes, THC peaks in THC mass transition eluted at 2.13, 2.74, 3.52, 5.51, 7.38, and 9.79 min were observed, suggesting that TDG can be hydrolyzed into TMG and THC (see Supplementary Information Figure S5C).

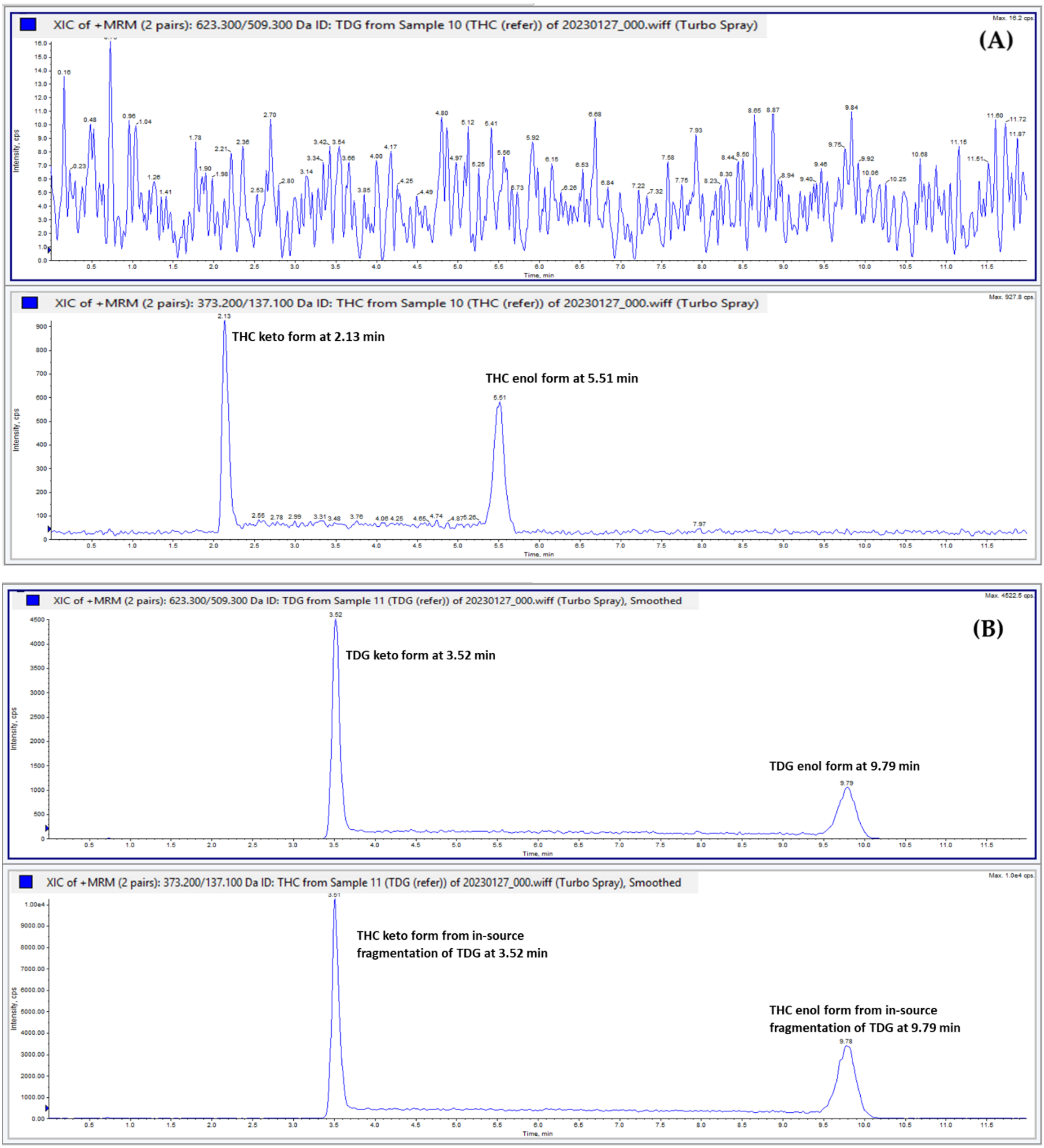

Figure S3. Representative chromatograms obtained from (**A**) THC and (**B**) TDG reference standards.

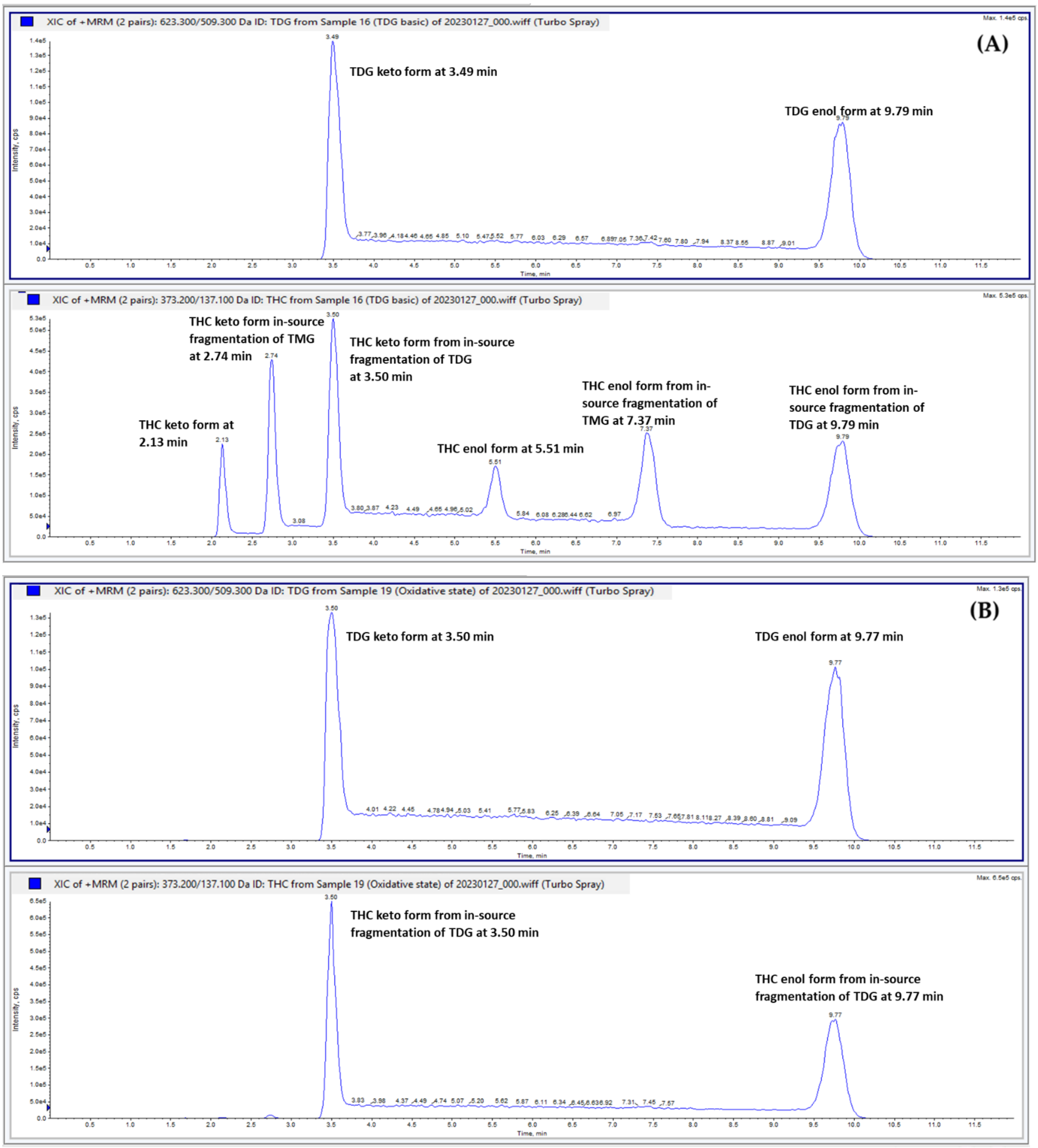

Figure S4. Representative chromatograms obtained from (**A**) basic hydrolysis and (**B**) oxidative stress of TDG reference standard.

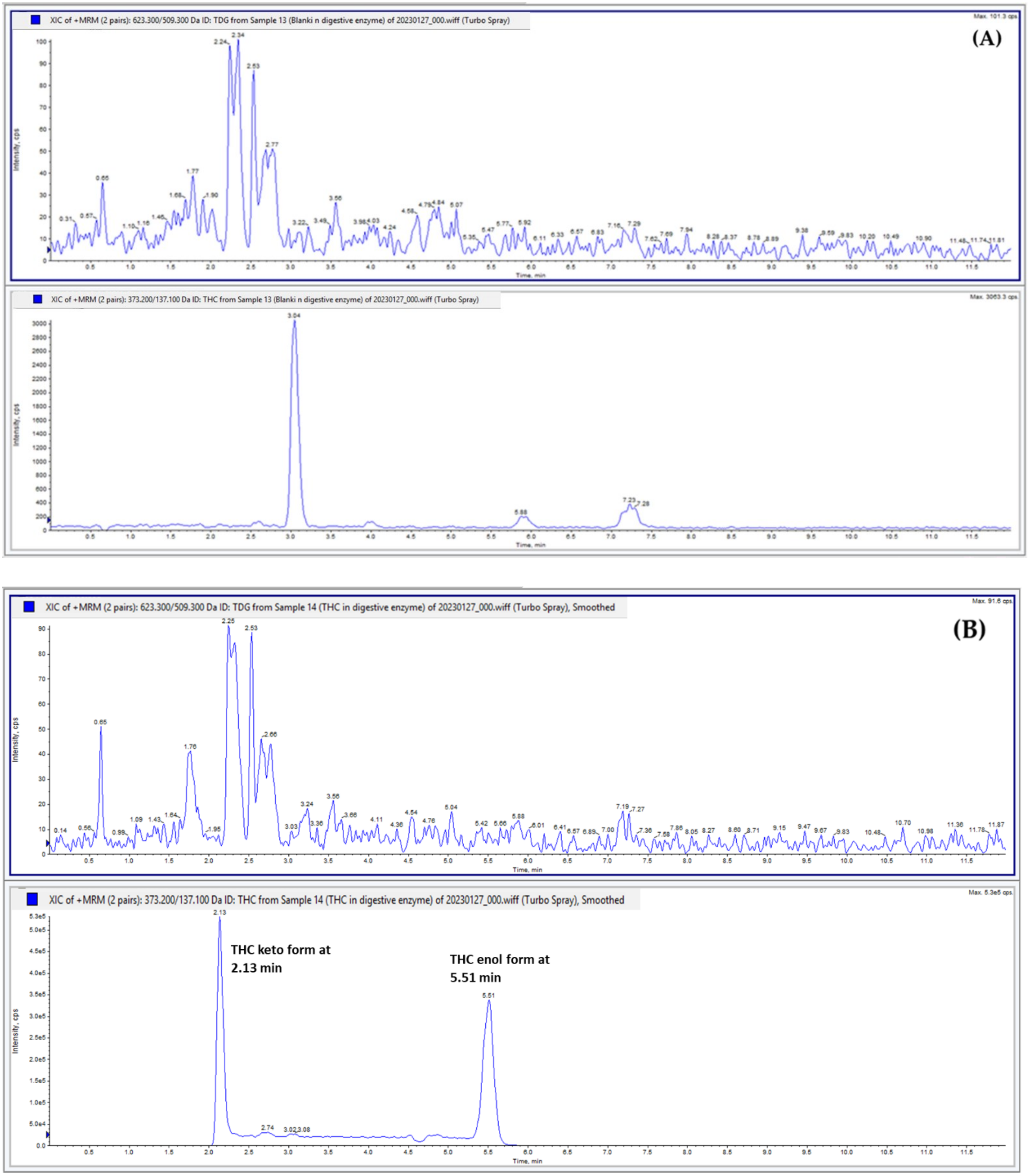
XIC of +MRM (2 pairs): 623.300/509.300 Da ID: TDG from Sample 13 (Blanki n digestive enzyme) of 20230127_000.wiff (Turbo Spray)
(A)
Intensity, cps
Time, min
XIC of +MRM (2 pairs): 373.200/137.100 Da ID: THC from Sample 13 (Blanki n digestive enzyme) of 20230127_000.wiff (Turbo Spray)
Max. 3063.3 cps
XIC of +MRM (2 pairs): 623.300/509.300 Da ID: TDG from Sample 14 (THC in digestive enzyme) of 20230127_000.wiff (Turbo Spray), Smoothed
Max. 91.6 cps
(B)
XIC of +MRM (2 pairs): 373.200/137.100 Da ID: THC from Sample 14 (THC in digestive enzyme) of 20230127_000.wiff (Turbo Spray), Smoothed
Max. 5.3e5 cps
THC keto form at 2.13 min
THC enol form at 5.51 min

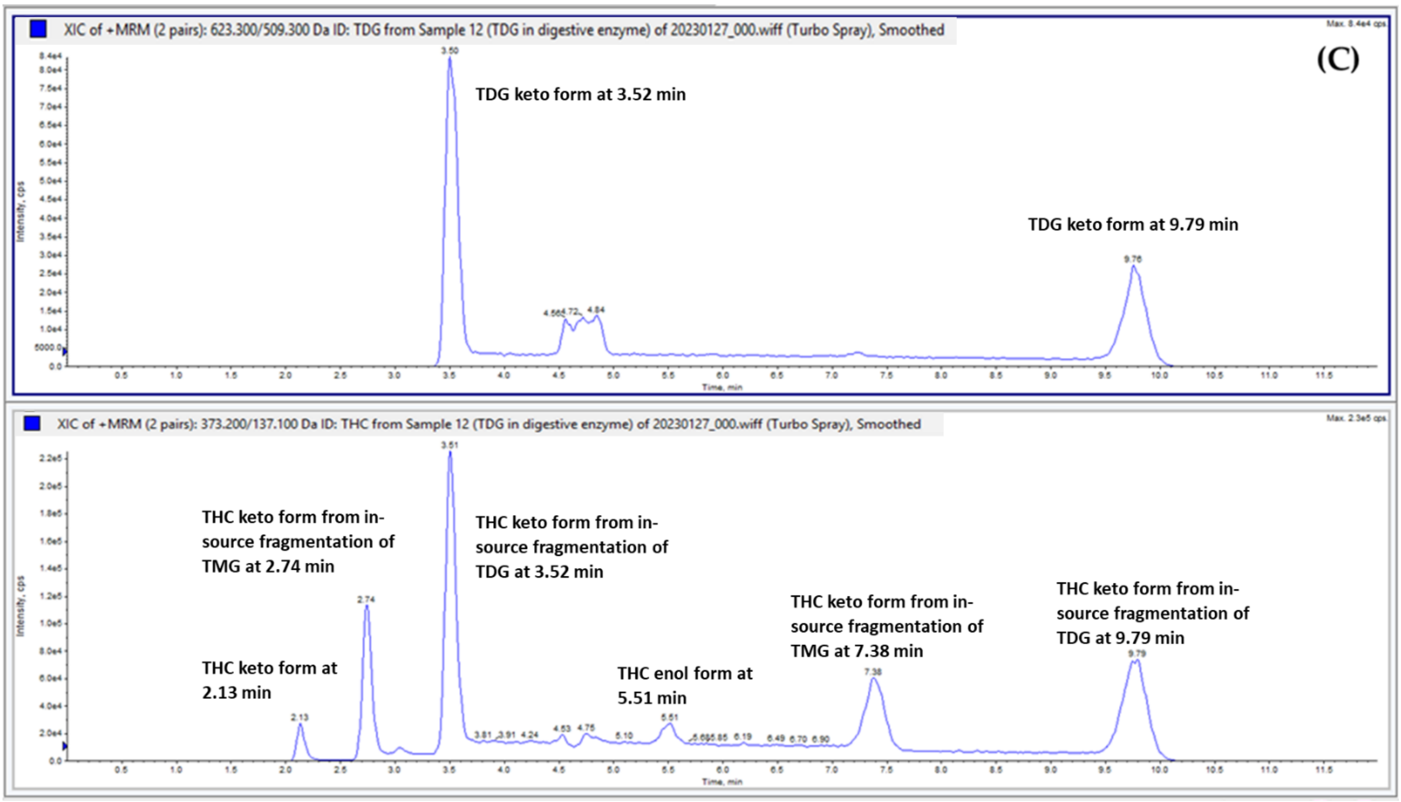

Figure S5. Representative chromatograms obtained from (**A**) blank digestive enzymes, (**B**) THC in digestive enzymes, and (**C**) TDG in digestive enzymes.

For unknown impurities identification, Q1 scan mode was applied to investigate unknown degradation products. According to the oxidative stress experiment, TDG was degraded to unknown degradation products eluted at retention times of 0.76 and 1.39 min before the THC keto peak (See Supplementary Information Figure S6). Mass spectra showed at *m*/*z* 132.1 and 145.3 [M+H$^+$] for the unknown peak at 0.76 min and *m*/*z* 114.4, 158.4, 262.7 [M+H$^+$] for the unknown peak at 1.39 min. Further MS fragmentation experiments are required to identify the unknown degradants.

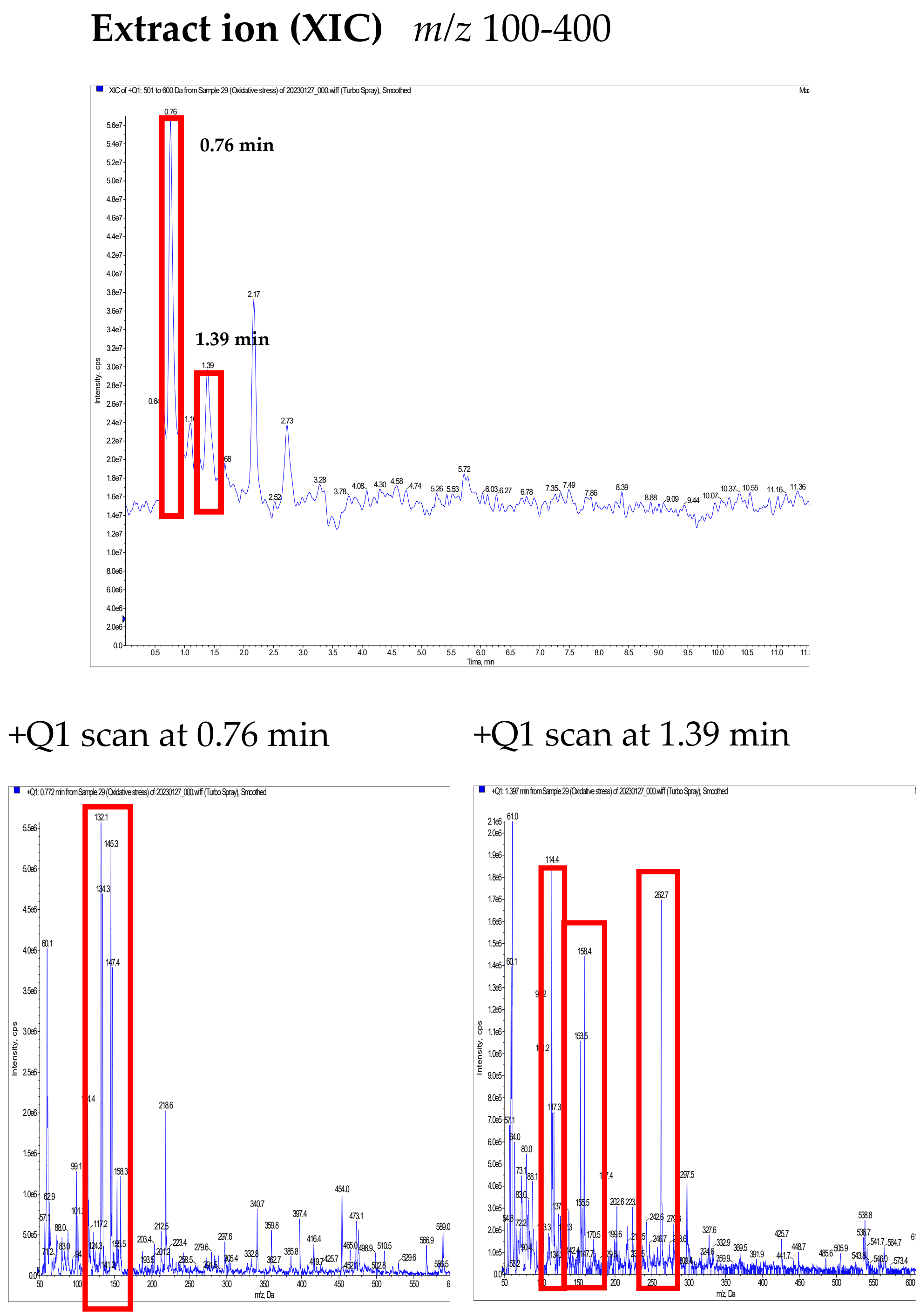

Figure S6. Representative chromatograms demonstrating the extract ion (XIC) at *m*/*z* 100-400 of the oxidative stress sample.
